# Differential Variation in Non-structural Carbohydrates in Root Branch Orders of *Fraxinus mandshurica* Rupr. Seedlings Across Different Drought Intensities and Soil Substrates

**DOI:** 10.3389/fpls.2021.692715

**Published:** 2021-12-08

**Authors:** Li Ji, Yue Liu, Jun Wang, Zhimin Lu, Lijie Zhang, Yuchun Yang

**Affiliations:** ^1^Jilin Academy of Forestry, Changchun, China; ^2^Key Laboratory of Sustainable Forest Ecosystem Management-Ministry of Education, School of Forestry, Northeast Forestry University, Harbin, China; ^3^School of Forestry, Shenyang Agricultural University, Shenyang, China

**Keywords:** non-structural carbohydrates, *Fraxinus mandshurica*, fine root, drought, soil substrate, root trait

## Abstract

Non-structural carbohydrates (NSCs) facilitate plant adaptation to drought stress, characterize tree growth and survival ability, and buffer against external disturbances. Previous studies have focused on the distribution and dynamics of NSCs among different plant organs under drought conditions. However, discussion about the NSC levels of fine roots in different root branch orders is limited, especially the relationship between fine root trait variation and NSC content. The objective of the study was to shed light on the synergistic variation in fine root traits and NSC content in different root branch orders under different drought and soil substrate conditions. The 2-year-old *Fraxinus mandshurica* Rupr. potted seedlings were planted in three different soil substrates (humus, loam, and sandy–loam soil) and subjected to four drought intensities (CK, mild drought, moderate drought, and severe drought) for 2 months. With increasing drought intensity, the biomass of fine roots decreased significantly. Under the same drought intensity, seedlings in sandy–loam soil had higher root biomass, and the coefficient of variation of 5th-order roots (37.4, 44.5, and 53% in humus, loam, and sandy–loam soil, respectively) was higher than that of lower-order roots. All branch order roots of seedlings in humus soil had the largest specific root length (SRL) and specific root surface area (SRA), in addition to the lowest diameter. With increasing drought intensity, the SRL and average diameter (AD) of all root branch orders increased and decreased, respectively. The fine roots in humus soil had a higher soluble sugar (SS) content and lower starch (ST) content compared to the loam and sandy–loam soil. Additionally, the SS and ST contents of fine roots showed decreasing and increasing tendencies with increasing drought intensities, respectively. SS and ST explained the highest degree of the total variation in fine root traits, which were 32 and 32.1%, respectively. With increasing root order, the explanation of the variation in root traits by ST decreased (only 6.8% for 5th-order roots). The observed response in terms of morphological traits of different fine root branch orders of *F. mandshurica* seedlings to resource fluctuations ensures the maintenance of a low cost-benefit ratio in the root system development.

## Introduction

In recent decades, forest decline and death caused by high temperatures and extreme droughts have occurred on a large scale worldwide (Allen et al., 2010; Choat et al., 2012; Zhang et al., 2015; Martínez-Vilalta et al., 2016). Global climate change is predicted to cause tree death and is becoming increasingly serious, which inevitably affects carbon metabolism and balance in the plant and changes its physiological metabolic functions (Choat et al., 2012; Adams et al., 2013). Non-structural carbohydrates (NSCs), as important substances involved in the life process of plants, are primarily composed of soluble sugar (SS) and starch (ST); they largely reflect the carbon supply status of plants and affect the growth and development of plants (Richardson et al., 2013). When plants undergo drought stress, the stored NSCs can be used as a buffer to temporarily supply plants for their growth and metabolism (Dietze et al., 2014). In recent years, the different responses of NSCs among different plant tissues or organs under drought stress have been discussed deeply (Martínez-Vilalta et al., 2016; Furze et al., 2019; Deng et al., 2020; He et al., 2020; Zhang et al., 2020). Many studies have found that the NSC concentration in root tissue is highest except for the trunk, which has an important impact on the storage and distribution of NSCs in trees (Dietze et al., 2014; Mei et al., 2015; Ji et al., 2020). However, the performance of NSCs with fine roots (especially different functional root sequences) in response to drought is ignored. Therefore, understanding the variation in the composition and level of fine root NSCs under drought conditions is of great significance for better recognition of the carbon balance and dynamics of plant survival and growth (Hartmann and Trumbore, 2016).

Accumulating evidence from field and laboratory experiments showed that root biomass usually decreased when plants were exposed to drought conditions (Meier and Leuschner, 2008; Eldhuset et al., 2013; Hertel et al., 2013; Zhou et al., 2018; Wang et al., 2021). However, Olmo et al. (2014) found that the fine root with diameters less than 0.5 mm would have increasing biomass under water deficit. The root system plays a crucial role in plant growth and productivity, especially in resource-constrained environments. The morphological and physiological plasticity of root systems reflects important mechanisms by which plants obtain limited soil resources (Ristova and Busch, 2014; Rogers and Benfey, 2015). Many researchers have demonstrated that changes in root ST under drought conditions are related to plant survival, and root NSC reserves play an important role in repairing embolism and preventing self-death (Rodríguez-Calcerrada et al., 2017; Kannenberg et al., 2018). Oswald and Aubrey (2020) observed that the root ST concentration of *Pinus palustris* in xeric environments increased delayed in summer compared with mesic habitats. ST stored in the root system can promote root growth and maintain root osmotic potential, ensuring that plants can absorb more water (Ge et al., 2012; Camarero et al., 2016). However, previous studies have mostly focused on the overall NSC level of the root system (including coarse roots and fine roots) (Hoch and Körner, 2003; Landhäusser and Lieffers, 2012; Hartmann et al., 2013; Yang et al., 2021), and the response of fine root NSCs with functional root branch orders to drought is rarely considered (Aubrey and Teskey, 2018; Nikolova et al., 2020). Fine roots (≤2 mm) are the main organ for water and nutrient absorption, and the most active and sensitive part of the root system (McCormack et al., 2015; Ma et al., 2018). Additionally, Pregitzer et al. (2002) found that the lower-order roots were produced later; the younger the age, the larger the contribution; the lower-order roots have higher nitrogen content and the respiration metabolism ability; and they are more sensitive and fragile than the higher-order roots. In addition, previous studies have mostly compared the effects of a single drought or severe drought on the root system, but gradient studies are lacking (Hartmann et al., 2013; Kannenberg et al., 2018; Blackman et al., 2019; Zhang et al., 2020). McDowell et al. (2008) speculated that trees depleted NSC only under mild or moderate drought, while severe drought caused the xylem to form embolisms while NSCs were not depleted. Recently, a meta-analysis based on 52 tree species around the world indicated that variations in plant NSCs were related to drought intensity, and the net loss of carbohydrates from roots was the most obvious (He et al., 2020). Therefore, it is of great significance to understand the response of fine root NSCs with branch root orders to different drought intensities.

Root traits play a vital role in the acquisition and transportation of water and nutrients, and they can strongly affect plant growth, survival, and response to climate change (Bardgett et al., 2014; Kong et al., 2014; Iversen et al., 2017). Most previous studies have demonstrated that the drought shapes thinner and deeper root systems to improve the ability to capture water and nutrients (Chapman et al., 2012). Zhou et al. (2018) based on 128 drought experiments found that the drought increased the specific root length (SRL) of woody plants by 30%, and there was a significant positive correlation between plant–SRL and drought. Although numerous studies have reported the response of root traits (high SRL, low diameter) to drought or water deficit (Comas et al., 2013; Fort et al., 2017; Zhou et al., 2018, 2019; Lozano et al., 2020; Nikolova et al., 2020), few studies have focused on the synergistic changes and relationships between root NSC levels and root traits under stress conditions (Ji et al., 2020; Yang et al., 2021). Olmo et al. (2014) studied the drought resistance response of 10 woody tree species seedlings and found that SRL increased significantly under drought conditions. In addition, the drought usually accompanied by increasing temperature induced the accumulation of ST in very fine (<0.5 mm) and fine roots (0.5–1 mm) (Di Iorio et al., 2016). It is a strategy that when water is limited, plants can build longer roots with less carbon. Indeed, increasing the carbon input per unit produces a larger surface area, length of fine roots, and more fine roots, which could facilitate optimization of the cost-benefit ratio of fine roots (Eissenstat et al., 2000; Ostonen et al., 2007). Under drought conditions, thicker roots with transport and storage functions tend to preserve NSCs (Konôpka et al., 2007; Yang et al., 2021), whereas thinner roots with absorption functions are severely affected by the drought (Olmo et al., 2014). Yang et al. (2021) found that a significant correlation between the root NSC concentration, root architecture, and SRL occurred in *Phyllostachys edulis* seedlings under drought conditions, indicating that the sensitivity of NSC concentration to drought supported the plasticity of root architecture to a certain extent, and plants could build low-cost roots through more carbon investment. In addition, when plants adapt to drought stress, they balance the lack of tissue radial growth by increasing the concentration of NSCs in the growing parts (Dietze et al., 2014; Kannenberg et al., 2018). Therefore, exploring the coupled relationship between the variation in fine root NSCs and root traits under drought conditions will help us to further understand the response strategies of plants to water deficit.

*Fraxinus mandshurica* Rupr. is one of the main timber species in northeastern China. In Jilin Province, China, *F. mandshurica* plantations are distributed in the north-south latitudes, and the soil types they inhabit are roughly divided into three categories, namely, humus soil, loam soil, and sandy–loam soil. Weemstra et al. (2017) found that the SRL and root tissue density (RTD) of the fine roots of European beech and Norway spruce were not significantly different in clay and sandy soil, but the dry mass of fine roots in sandy soil (both species) was 10 times that in clay. Paudel et al. (2016) studied these interactions in the osmometer of an orchard and clay sandy–loam and found that soil type significantly affects the morphological traits of all root branch orders of *Citrus paradisi* Macf. The interaction of the root system with soil quality and water will cause changes in root growth, structure, and function (Paudel et al., 2016). To our knowledge, few studies have focused on the response of fine root NSCs to drought intensity gradients in different soil substrates, and even less is known regarding the coupled relationship between fine root NSCs and traits. In this study, 2-year-old *F. mandshurica* potted seedlings with different drought intensities and soil substrates were set up, and the variation and the coupled relationship of the fine root traits and NSC content of different root branch orders of *F. mandshurica* seedlings under different water and soil conditions were compared. It was hypothesized that (1) given different physicochemical properties, seedlings planted among different substrates will have variable root traits; drought will significantly change root traits to increase the ability to capture water, e.g., increase SRL and decrease diameter; (2) with the increase of drought intensity, the fine roots of seedlings subjected to drought stress will consume SS and accumulate ST content to survival under carbon-limited conditions; and (3) due to the trade-off between plant carbon investment and growth under drought conditions, the variation in fine root NSCs will be closely related to root traits.

## Materials and Methods

### Experimental Site and Sapling Preparation

A controlled pot experiment was conducted at Xinli Town, Jingyue Development District, Changchun, which is located in Jilin Province, China (43°33′ N–44°41′ N, 125°19′ E–125°24′ E). The location has a temperate continental monsoon climate with a frost-free period of 140 days; and with a mean annual rainfall of 600–800 mm, which mainly falls from July to September and a mean annual temperature of 4.6°C.

The 2-year-old *F. mandshurica* Rupr. seedlings, of the Hongwei nursery of Lushuihe Forestry Bureau in Jilin Province, were used as experimental materials. They were transplanted into plastic pots (24 cm × 20 cm) in April 2017 and were placed in flat, open canopies before they started to bud. An equal number of pots were filled with humus, loam, and sandy-loam soil, respectively. The humus soil and loam soil were collected in a secondary forest (consisting of *F. mandshurica*, *Juglans mandshurica*, *Phellodendron amurense*, *Picea asperata*, and *Larix olgensis*) in the Lushuihe Forestry Bureau. The main soil type is a Eum-Orthic Anthrosol according to the Food and Agricultural Organization soil classification system. The sandy-loam soil consists of a mixture of equal volumes of loam (above mentioned) and sand. All potted seedlings were placed under a rain shelter (consisting of iron shelves and thick plastic). The soil surface was well ventilated throughout the experiment. For the experimental area, the pots were placed in rows 50 cm apart from the neighbors under full sunlight. They were kept well-watered prior to the application of drought treatments, and the gravimetric soil water content was initially maintained at field capacity. Fertilizer was not added during the experimental period.

### Experimental Design and Sampling

Before the beginning of the drought experiment, three soil substrate samples were collected in July 2017 to determine the soil total nitrogen, total phosphorus, available phosphorus and soil physical structure, water content, and field water holding capacities. The field water holding capacity of humus, loam, and sandy-loam soil was 54.6, 36.4, and 20%, respectively. About 120 pots of cultured *F. mandshurica* seedlings (relatively uniform seedlings) were randomly selected for three different substrates, 40 pots per substrate, respectively. The basal diameter and height of each seedling were measured using a Vernier caliper with an accuracy of 0.01 mm and a tape measure with an accuracy of 0.1 cm prior to the application of drought treatments in July 2017, respectively (Supplementary Table 1).

The soil substrates and drought were conducted for a two-factor experiment. Three soil substrates were set with four water available gradients, the control (CK): approximately 80–85% of the maximum field water-holding capacity (FC); mild drought (T1): 60–65% of the FC; moderate drought (T2): 40–45% of the FC; and severe drought (T3): 20–25% of the FC. About 10 seedlings were assigned to each treatment, totaling 120 seedlings (10 seedlings × four treatments × three soil substrates). Briefly, the soil moisture content of the pots was monitored by weight. The pots were weighed before the progressive drought experiment, and then, the theoretical weights for different drought intensities of the three substrates were calculated. These pots were thereafter controlled daily to maintain a constant weight until the experiment ended in September 2017. A detailed description of drought control was provided in a previously conducted study (Ji et al., 2020). All seedlings were not fertilized before and during the drought experiment.

In each experimental treatment, the roots of 10 seedlings were destructively sampled after 2 months of continuous drought stress. Roots were sorted carefully out of the soil, and root samples were washed free of soil particles with deionized water until the branching structure of the roots could be identified, and then put into the labeled pocket and stored in a portable refrigerator (2–3°C). Then, the root samples were divided into two parts: (1) root morphology analysis and biomass sample; (2) chemical properties analysis sample (for root chemical traits and NSCs). All samples were shipped back to the laboratory on the same day and stored in a freezer at −20°C. In this study, only live root samples were measured, and the dead roots were picked and discarded.

### Soil Physicochemical Property

The soil physical and chemical properties of the three soil substrates were determined before drought stress (Table 1). The soil total nitrogen was determined by the Kjeldahl titration. The soil total phosphorus was determined by the sulfuric acid-perchloric acid-molybdenum anti-colorimetric method (Yang et al., 2018). Soil available phosphorus was extracted by double acid extraction, soil water content, and bulk density were determined by the ring knife method (Yang et al., 2018), and soil total porosity, aeration porosity, water absorption multiple, water seepage rate, and evaporation rate were measured by following the description of Wei et al. (2015).

**TABLE 1:** Physicochemical properties of three soil substrates.

Soil property	Humus soil	Loam soil	Sandy–loam soil
Bulk density (g cm^–3^)	1.18 ± 0.02b	1.35 ± 0.02a	1.32 ± 0.02a
Total porosity (%)	53.19 ± 1.12b	58.90 ± 0.91a	36.72 ± 0.95c
Aeration porosity (%)	25.61 ± 0.48a	23.52 ± 0.53a	13.01 ± 0.99b
Water absorption capacity	0.23 ± 0.01b	0.26 ± 0.01a	0.18 ± 0.01c
Penetrate rate (g min^–1^)	4.49 ± 0.04a	4.01 ± 0.12b	3.04 ± 0.18c
Evaporation rate (g h^–1^)	0.59 ± 0.01a	0.50 ± 0.01b	0.37 ± 0.01c
Total nitrogen (mg g^–1^)	7.09 ± 0.78a	3.11 ± 0.05b	1.20 ± 0.03c
Total phosphorus (mg g^–1^)	0.71 ± 0.04a	0.34 ± 0.09b	0.32 ± 0.01b
Available phosphorus (mg kg^–1^)	13.10 ± 0.82a	5.04 ± 0.21b	12.75 ± 0.69a

*The different letters in the same line indicate significant differences among the different treatments (p < 0.05).*

### Fine Root Morphological and Chemical Traits

In the laboratory, root samples for morphological analysis were carefully dissected with forceps on the basis of branch order, following the procedure described in Pregitzer et al. (2002) and Wang et al. (2006), with the distal non-woody roots regarded as first-order roots (1st-order root), and the next root segment was the 2nd-order roots. For the 4th- and 5th- order roots, root axes larger than 2 mm were separated from each other where necessary with scissors. Then, root samples were scanned with an Expression 10000XL 1.0 scanner at Northeast Forestry University (Epson Telford Ltd., Telford, United Kingdom). The average diameter, total length, and volume of root tips were determined with the root system analyzer software (WinRhizo 2004b, Regent Instruments, Inc., Québec, QC, Canada). These root samples were oven-dried at 65°C to determine the constant weight (nearest 0.0001 g) for fine root biomass and the SRL, specific root surface area (SRA), and RTD were calculated.

For root chemical analyses, a part of the root of the seedlings was classified quickly, and then, the fine root samples were placed at 65°C and dried for 48 h to constant weight. The dried fine root sample was ground and homogenized by using a ball mill instrument (RETSCH MM 400, Haan, Germany), and 1 g of the dry powder sample was weighed and pressed with an FYD-20 electric tableting machine (Nuoleixinda Technology Co., Ltd., Tianjin, China) to a boat with a thickness of 6 mm and a diameter of approximately 13 mm. For the pellet sample, the tableting conditions can be adjusted according to the actual conditions. The tableting conditions of the test were maintained at a pressure of 16 MPa for 3 min. The carbon, nitrogen, and phosphorus elements in the root samples after tableting were measured by a J200 Tandem laser spectroscopic element analyzer (Applied Spectra, Inc., Fremont, CA, United States).

### Fine Root Non-structural Carbohydrate Concentration

The NSC concentration was defined as the sum of SS and ST concentrations that were measured using the anthrone method (Yemm and Willis, 1954). Root samples (0.1000 g) were placed into a 10 ml centrifuge tube, and 2 ml of 80% ethanol was then added. The mixture was incubated at 80°C in a shaking water bath for 30 min and then centrifuged at 4,000 rpm for 5 min. Two additional extractions from the pellets were carried out with 80% ethanol. The supernatant was retained, combined, and stored at −20°C for SS determination.

Starch was extracted from the ethanol-insoluble pellet after ethanol was first removed by evaporation. The ST in the residue was then released by boiling in 2 ml distilled water for 15 min. After cooling to room temperature, 2 ml of 9.2 M HClO_4_ was added, and the mixture was shaken for 15 min. About 4 ml of distilled water was then added, and the mixture was centrifuged at 4,000 rpm for 5 min. A further extraction was carried out with 2 ml 4.6 M HClO_4_. The supernatant was also retained, combined, and stored at –20°C for ST determination.

The soluble sugar and ST determination were performed based on the absorbance at 625 nm using the same anthrone reagent in a spectrophotometer (Yemm and Willis, 1954). Sugar concentration was calculated from the regression equations based on glucose standard solutions and ST concentration by multiplying glucose concentration with a conversion factor of 0.9 (Osaki et al., 1991).

### Data Analysis

Normality and variance homogeneity requirements were met, and no data transformation was necessary. The effects of drought intensity, soil substrate, and root order as fixed factors on the fine root NSC concentration and morphological traits were tested using the three-way ANOVA by SPSS 19.0 (IBM Corp., Armonk, NY, United States). The differences in fine root traits of seedlings between different treatments were examined (Turkey’s test, α = 0.05); redundancy analysis (RDA) was performed on the fine root traits of seedlings under different treatments using the Canoco software (Version 4.56, Biometris Plant Research International, Wageningen, Netherlands). The Monte Carlo test was performed on the parameters in the RDA using R software (vegan package) (R Core Team, 2018). A multiple linear regression analysis was performed using the Sigmaplot 12.5 software (Systat Software Inc., San Jose, CA, United States) to analyze the influence of root traits on the fine root NSC (SS and ST) content in all treatments. All data are mean ± SE. All bar figures were drawn using Origin Pro 8.5 (OriginLab, Northampton, MA, United States).

## Results

### Fine Root Biomass and Morphological Traits Among Root Branch Orders

**FIGURE 1:**
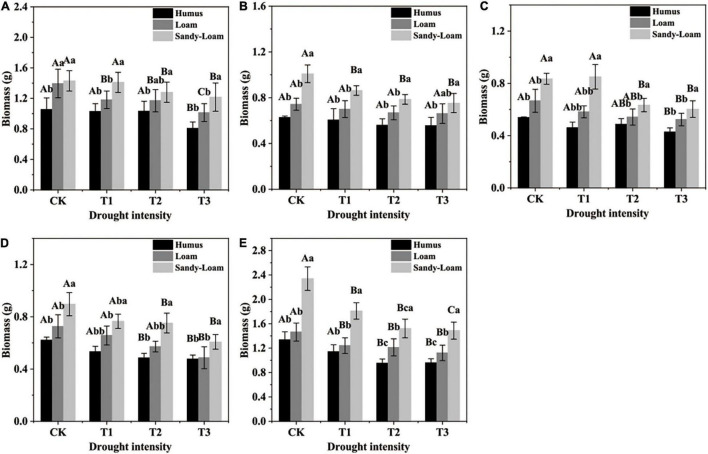
The fine root biomass of *Fraxinus mandshurica* seedlings in different drought intensities and soil substrates. The different lowercase letters denote significant differences among soil substrates (*p* < 0.05). The different uppercase letters denote significant differences among drought intensities (*p* < 0.05). **(A)** 1st-order root; **(B)** 2nd-order root; **(C)** 3rd-order root; **(D)** 4th-order root; **(E)** 5th-order root. CK: control; T1: mild drought; T2: moderate drought; T3: severe drought.

Soil substrates, drought intensity, and root order had significant effects on the fine root biomass of *F. mandshurica* seedlings (Supplementary Table 2). With the increase in drought intensity, the fine roots biomass in the three substrates showed a progressive decrease (Figures 1A–E). The biomass of fifth-order roots had the highest coefficient of variation (among different drought intensities), which was 37.4, 44.5, and 53% in humus, loam, and sandy-loam soil, respectively (Figure 1E). Under the same drought treatment, the fine root biomass with all branch orders was the highest in sandy-loam soil and the lowest in humus soil. With the increase in drought intensity, the coefficient of variation of the fifth-order root biomass among different soil substrates was the highest, which was 61.8% (in CK), 36.6% (in T1), 57% (in T2), and 40.4% (in T3) (Figure 1E).

**FIGURE 2:**
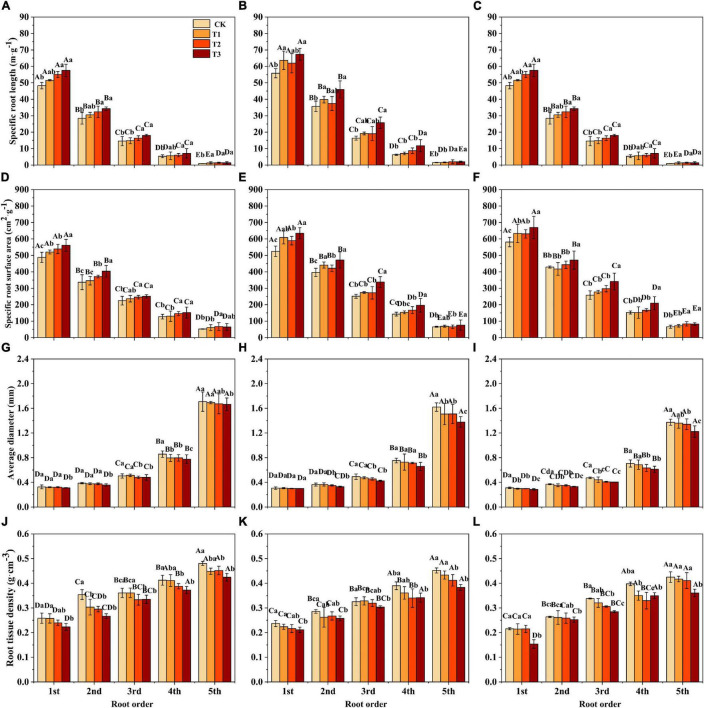
The specific root length (SRL) **(A–C)**, specific root surface area (SRA) **(D–F)**, the average diameter (AD) **(G–I)**, and root tissue density (RTD) **(J–L)** of *Fraxinus mandshurica* seedlings in different drought intensities and soil substrates. The different lowercase letters denote significant differences among drought intensities (*p* < 0.05). The different uppercase letters denote significant differences among root branch order (*p* < 0.05). **(A,D,G,J)** Humus soil; **(B,E,H,K)** loam soil; **(C,F,I,L)** sandy-loam soil; CK: control; T1: mild drought; T2: moderate drought; T3: severe drought.

Soil substrate, drought intensity, and root order had significant effects on the SRL, SRA, and RTD of *F. mandshurica* seedlings (Supplementary Table 2). Under the same drought intensity, the SRL and SRA of all root branch orders in the humus soil were the lowest and showed a significant decrease with increasing root order (Figures 2A–F). The average diameter (AD) and RTD of all root branch orders were the highest and lowest in the humus and sandy-loam soil, respectively, and showed a significant increasing trend with ascending root order (Figures 2G–I,J–L). With the increase in drought intensity, the SRL and SRA of seedlings for all soil substrates increased significantly. Compared with CK, the SRL and SRA under T3 treatment increased significantly by 39% (variation range 19.1–88.6%) and 22.3% (variation range 9.9–37.4%), respectively. Compared with CK, the RTD of seedlings in all three soil substrates was the lowest under T3 treatment, especially in the 1st-root order, which decreased by 13.7% (humus soil), 10.7% (loam soil), and 28.6% (sandy-loam soil) (Figure 2J). The AD of all root branch orders was less affected by drought intensity (Figures 2G–I and Supplementary Table 2).

### Fine Root Chemical Traits Among Root Branch Orders

**FIGURE 3:**
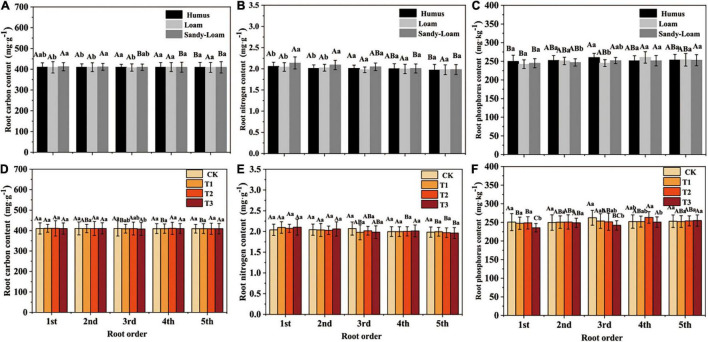
The root carbon **(A,D)**, nitrogen **(B,E),** and phosphorus **(C,F)** of *Fraxinus mandshurica* seedlings in different soil substrates **(A–C)** and drought intensity **(D–F)**. The different lowercase letters denote significant differences among drought intensities (*p* < 0.05). The different uppercase letters denote significant differences among root branch order (*p* < 0.05).

The fine root carbon, nitrogen, and phosphorus contents of *F. mandshurica* seedlings were significantly different among different root branch orders. With increasing the root order, the fine root carbon and nitrogen contents showed a decreasing trend, whereas the fine root phosphorus content showed an increasing trend (Supplementary Table 3 and Figures 3A–F). The soil substrate had a significant effect on the fine root carbon and nitrogen contents of *F. mandshurica* seedlings (Supplementary Table 3). The carbon and nitrogen contents of all root branch orders in sandy-loam soil were significantly higher than those of humus soil. The carbon and nitrogen contents of 1st-order roots in sandy-loam soil were the highest, which were 412.1 and 2.14 mg g^–1^, respectively (Figures 3A,B). The drought had a significant effect on the fine root carbon and phosphorus content of *F. mandshurica* seedlings, and the phosphorus content of the first fourth root order under the T3 treatment was lower than that of the CK (Figure 3F).

### Fine Root Non-structural Carbohydrate Content Among Root Branch Orders

**FIGURE 4:**
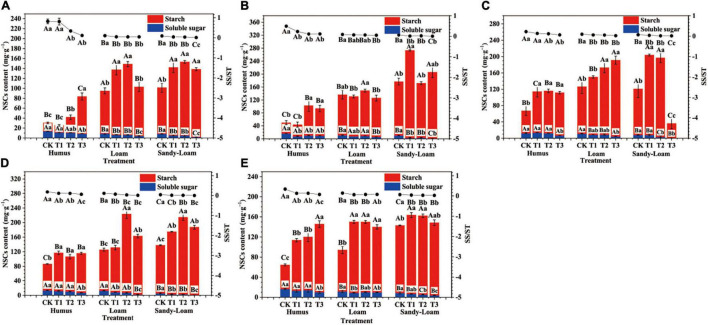
The fine root non-structural carbohydrate (NSC) content of *Fraxinus mandshurica* seedlings in different drought intensities and soil substrates. The histogram represents the soluble sugar (SS) and starch (ST) content, and the line chart represents the SS-ST ratio (SS/ST). The different lowercase letters denote significant differences among soil substrates (*p* < 0.05). The different uppercase letters denote significant differences among drought intensities (*p* < 0.05). **(A)** 1st-order root; **(B)** 2nd-order root; **(C)** 3rd-order root; **(D)** 4th-order root; **(E)** 5th-order root. CK: control; T1: mild drought; T2: moderate drought; T3: severe drought.

Soil substrate, drought intensity, and root order had significant effects on the SS, ST, and total NSC content of the fine roots of *F. mandshurica* seedlings (Supplementary Table 4). Under the same drought treatment, the SS content of seedlings in humus soil was the highest for all root branch orders. With ascending root order, the SS content of fine roots of seedlings in humus soil was 107.9, 162.7, 125.7, 269.2, and 118.5% higher than those of sandy-loam soil, respectively (after the average intensity of the four droughts) (Figures 4A–E). In all soil substrates, the SS content of the fine roots decreased with the increasing drought intensity. With increasing root order, the SS content of fine roots under the T3 treatment was 51.3, 58.1, 62.1, 68.7, and 36.5% lower than that of CK, respectively (after the average of the three soil substrates) (Figures 4A–E). Under the same drought intensity, seedlings in sandy-loam soil had the highest ST and total NSC contents for all root orders. The ST and total NSC contents of lower-order roots in different soil substrates varied greatly. The ST and total NSC content of the 1st-order and 2nd-order roots in sandy-loam soil were 276.1, 231.1, 195.8, and 186.7% higher than those of humus soil, respectively (after averaging the intensity of the four droughts) (Figures 4A–E). In all soil substrates, the fine root ST content generally increased with increasing drought intensity. The ST and total NSC contents of the 1st-order roots under the T3 treatment were 58.6 and 43.4% higher than those of the CK, respectively (Figure 4A).

### Relationship Among the Fine Root Biomass, Traits, and Non-structural Carbohydrate Content

**FIGURE 5:**
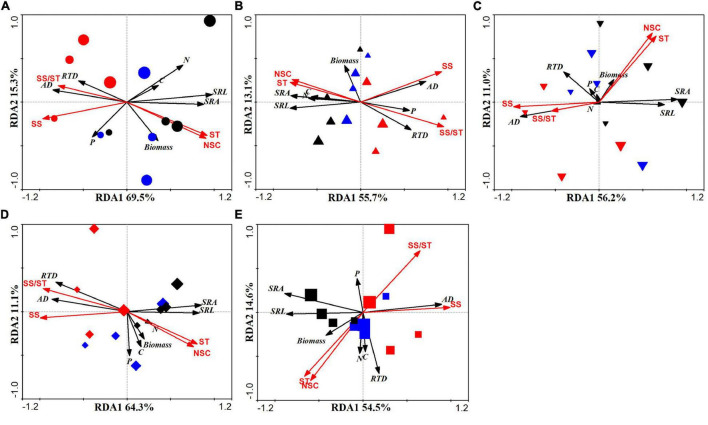
Redundancy analysis of fine root morphological and chemical traits in different drought intensity and soil substrates. **(A)** 1st-order root; **(B)** 2nd-order root; **(C)** 3rd-order root; **(D)** 4th-order root; and **(E)** 5th-order root. Red symbols, humus soil; blue symbols, loam soil; black symbols, sandy-loam soil. SRL: specific root length; SRA: specific root surface area; AD: average diameter; RTD: root tissue density; SS: soluble sugar; ST: starch; C: root carbon; N: root nitrogen; P: root phosphorus. The greater symbols indicate the stronger drought intensity.

The root morphological and chemical traits of the first five orders of roots of *F. mandshurica* seedlings under different drought intensities and soil conditions were analyzed by RDA, and the results showed that the first two axes of the RDA explained approximately 65% of the total variation between all treatments (Figures 5A–E). The first and second ordination axes indicated the variations in fine root morphological traits, chemical traits, and biomass, respectively. Soil substrates and drought intensity in plots had a good degree of separation. The fine root morphological and chemical traits under all treatments were subjected to partial Monte Carlo tests. For the 1st-order roots, SS and ST exhibited the highest degree of the total variation in fine root traits, which were 32 and 32.1%, respectively (Supplementary Table 5). With increasing root order, the variation in root traits under ST decreased only 6.8% (for 5th-order roots) (Supplementary Table 5).

**FIGURE 6:**
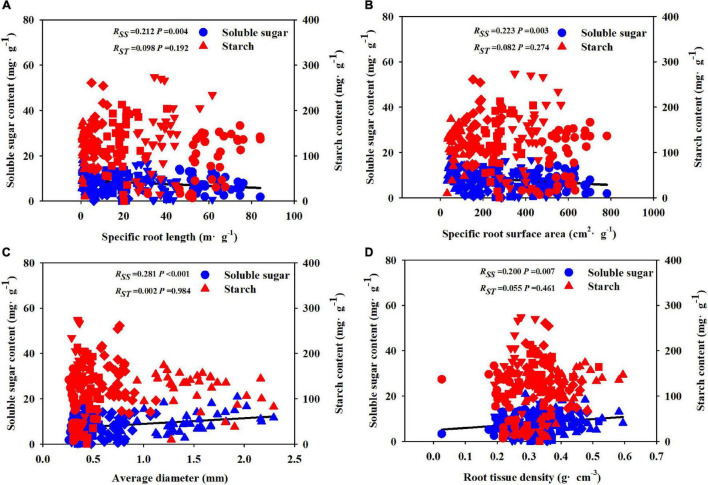
Relationships between soluble sugar, starch concentration, and fine root morphological traits [SRL **(A)**, SRA **(B)**, AD **(C)**, and RTD **(D)**] during the whole experiment period. R: correlation coefficient; SS: soluble sugar; ST: starch; SRL: specific root length; SRA: specific root surface area; AD: average diameter; RTD: root tissue density; circle represents 1st-order root; down-triangle represents 2nd-order root; square represents 3rd-order root; diamond represents 4th-order root; up-triangle represents 5th-order root.

The Pearson correlation analysis showed that under different soil substrates and drought treatments, the first five orders of root morphological and chemical traits were significantly correlated with SS, SRL, and SRA were significantly negatively correlated with SS, whereas the AD and RTD were significantly positively correlated with SS (Figures 6A–D).

## Discussion

Our results highlighted several key findings related to NSC and fine root traits of *F. mandshurica* seedlings under different soil substrates and drought intensities. First, with the increase of drought intensity, the fine root biomass decreased significantly. Under the same drought intensity, there was higher biomass in relatively barren sandy-loam soils, and the coefficient of variation for fifth-order roots was higher than those of lower-order roots. Second, compared with chemical traits, the morphological traits of fine roots were more sensitive to soil substrates and drought intensities. With increasing drought intensity, the SRL and AD of all root orders increased and decreased, respectively. Finally, the fine roots in the humus soil had higher ST content and lower ST content. With the increase of drought intensity, the SS and ST content of the fine roots showed decreasing and increasing trends, and the NSC content of the fine roots significantly correlated with root morphological traits. The variation in fine root NSC content in different soil conditions was supported to some extent by the plasticity of root morphology.

### Response of Root Morphology and Biomass to Drought and Soil Substrate

In this study, the fine root biomass of seedlings in sandy-loam soil (soil nutrient relatively poor) was significantly higher than that in humus soil (soil nutrient relatively rich), which was consistent with those of previous studies (Hertel et al., 2013; Poorter and Ryser, 2015). Weemstra et al. (2017) demonstrated that the fine root mass and the growth rate of fine roots of *Fagus sylvatica* and *Picea abies* in sandy soil were three times and 10 times higher than those of clay soil, respectively. In response to increased nitrogen availability, Hendricks et al. (1993) proposed two hypotheses for fine roots: (1) a decrease in carbon allocation and an unchanged turnover rate, or (2) an unchanged carbon allocation and the increase in the turnover rate. For both cases, a decrease in fine root biomass was expected. Liu et al. (2020) found that the higher-order root biomass of *Pinus tabuliformis* seedlings under the 20% field water holding capacity treatment was 33.3% lower than that under the 80% field water holding capacity treatment. It was found that the biomass of higher-order roots (e.g., fifth-order) had the highest degree of variation under different soil substrates and drought treatments, which was due to the different responses of fine roots of different root sequences to resource changes (Withington et al., 2006; Guo et al., 2008).

The morphological traits of fine roots, such as AD, SRL, and RTD, are important functional parameters that characterize or affect the water absorption efficiency and ability of roots. Generally, fine roots with smaller diameters and larger root lengths have higher water absorption efficiency (Freschet and Roumet, 2017; Dhiman et al., 2018; Ma et al., 2018). Root morphological characteristics responded to drought here in accordance with our first hypothesis as well, that is the root system of seedlings in humus soil had lower SRL, lower SRA, higher AD, and RTD, which is consistent with the research of other scholars on the fine root traits of *Pinus tabulaeformis*, *L. olgensis*, and *F. mandshurica* with increased nutrient availability (Liu et al., 2009; Wang et al., 2013). Gruber et al. (2013) believed that soil nitrogen deficiency usually promoted the elongation of main roots and some lateral roots. Plants can increase the absorption capacity of the root system in two different ways to adapt to water or nutrient shortages, (1) increase root yield and maintain a larger absorption surface area (acquisition strategy), or (2) increase the efficiency per unit mass by changing the morphology and physiological condition of the root system (conservative strategy) (Lõhmus et al., 2006; Ostonen et al., 2007). In this study, with the increase of drought intensity, the SRL and SRA of the first five order roots increased significantly, and the RTD decreased significantly, this is a manifestation of *F. mandshurica* seedlings coping with water-deficit conditions. In addition, the results showed that the differences in various morphological indicators between root orders had reached a significant level, indicating that the fine roots of *F. mandshurica* had a high degree of morphological heterogeneity, which was consistent with the previous study of the plasticity of the morphological traits of the root system from the perspective of orders (Pregitzer et al., 1997, 2002; Wang et al., 2006). Cortina et al. (2008) found that *Pistacia lentiscus* seedlings could rapidly expand the length and surface area of fine roots when exposed to drought stress, and shaped fine roots to avoid damage to arid environments. Generally, the larger the SRL or SRA and the smaller the AD, the higher the water absorption efficiency of fine roots (Freschet and Roumet, 2017; Dhiman et al., 2018). The results of this study emphasized the adaptation strategies of the fine roots of *F. mandshurica* seedlings in three soil substrates to different drought intensities.

### Effects of Drought and Soil Substrate on Root Non-structural Carbohydrate

Soluble sugar is an important osmotic adjustment substance for plants to tolerate arid environments. It can reflect the drought status of plants. Changes in its concentration can adjust the osmotic pressure of cells in plants to maintain normal physiological activities to adapt to drought stress (Quentin et al., 2015). In this study, compared with sandy-loam soil, the SS content of fine roots (especially lower-order roots) in humus soil was significantly increased, the ST was significantly decreased, the NSC was significantly reduced, and the SS-to-ST ratio was significantly increased. The development of fine roots accelerates the consumption of NSCs, and the roots decrease the ratio of SS to ST to cope with stress conditions. The increase in the ratio is beneficial for plants to adjust osmotic potential to maintain the transport channel between leaves and roots, and improve water transport efficiency (Sala et al., 2012). Some studies have pointed out that lower-order roots may obtain greater carbon investment than higher-order roots because the main function of lower-order roots is to absorb water and nutrients (absorptive roots), whereas higher-order roots are responsible for transporting nutrients and supporting the entire root (Liu et al., 2020). The results showed that after 2 months of drought, the lower-order roots of *F. mandshurica* seedlings had more NSC content than the higher-order roots. This result implied that in the case of limited carbon, *F. mandshurica* preferentially allocated carbon to thin roots rather than thick roots, which was similar to the research results in hybrid poplar *Populus* × *canadensis* cv. *Eugeneii* (Kosola et al., 2001) and *P. tabuliformis* (Liu et al., 2020). Di Iorio et al. (2016) found that the ST content of very fine root (<0.5 mm) of *F. sylvatica* saplings in warming and drought treatment was significantly higher than that in warming conditions. Under carbon-limiting conditions, plants prioritize the distribution of fine roots. They could reduce carbon consumption (higher-order roots consume more carbon than lower-order roots). However, maintaining fine roots could absorb more water and nutrients (Finér et al., 2011). Liu et al. (2020) conducted short-term drought stress on *P. tabuliformis* seedlings and found that the amount of ^13^C allocated to the first three roots 120 days after isotope labeling was significantly higher than that of the control in the moderate and mild drought, whereas the amount of ^13^C allocated to fifth-order roots was significantly higher in the control. Some studies have suggested that carbon starvation caused by drought stress may only exist in belowground organs and has little to do with the aboveground parts. Specifically, thick roots are essential to alleviate the decline in the NSC content of the entire plant caused by drought (Hartmann et al., 2013; Kannenberg et al., 2018), and drought will cause the loss of phloem function, making the aboveground and underground parts uncoupled. When plants are subjected to drought stress, the NSC content of the aboveground organs will increase or remain stable for a short time, whereas the NSC content of the roots will decrease significantly (Sevanto et al., 2014).

Galvez et al. (2011) found that drought significantly increased the concentrations of SS and ST in the fine roots of *Populus tremuloides* seedlings. The results of this study supported the second hypothesis, with the increase in drought intensity, the variation in root NSCs was higher than that of root chemical traits, root SS content decreased, and ST and total NSC contents increased. A possible explanation for this might be that the path of NSCs to the fine roots of *F. mandshurica* seedlings was blocked after 2 months of drought stress, causing the carbohydrates produced by leaf photosynthesis unable to transport to the belowground organs. Fine roots could only rely on regulating their own NSC levels to cope with drought stress (Dietze et al., 2014). However, the inference presented in this study needs to be further verified by combining the dynamic changes in fine root NSC content under different periods of drought treatment. The results revealed the interaction of different soil substrates and drought intensities on the variations in the first five root order NSCs of *F. mandshurica* seedlings.

### Linking the Fine Root Traits to the Non-structural Carbohydrate Level

The root system is the organ responsible for absorbing water and is the first responder to a variety of stresses. Because roots tend to grow in moist soil, the impact of water shortages can be minimized (Brunner et al., 2015; Weemstra et al., 2016). Under drought stress conditions, more carbohydrates are allocated to lower-order roots to promote root structure and growth (Liu et al., 2020). Although there is evidence that thick roots increase NSC accumulation under drought conditions (Yang et al., 2016), whether the variation in fine root traits is directly related to NSC accumulation remains to be explored. Previous research revealed that the root tip length and diameter of the seedlings (three broad-leaved tree species in the temperate zone) under drought conditions explained the higher variation in ST and SS contents (Ji et al., 2020). In this study, drought reduced the root biomass of *F. mandshurica* seedlings, changed the root traits of all root orders, and promoted the absorption of water and nutrients. This response may be related to the increased demand for osmotically active C compounds under drought conditions. NSCs can provide fuel for root respiration and are an important substrate for root growth and physiological regulation (George et al., 2003; Xu et al., 2008). In this study, SRL and SRA of all samples were significantly negatively correlated with the SS content of fine roots, and AD and RTD were significantly positively correlated with the ST content, which confirms the third hypothesis. When the root diameter is smaller, the level of NSC required to build and maintain a thin root per unit length of the plant would be lower than that of the thicker root (Guo et al., 2004). Ma et al. (2018) pointed out that the root diameter of herbaceous plants was thinner than that of woody plants, so more carbon could be easily distributed to the roots under drought conditions. Maguire and Kobe (2015) pointed out that drought stress may directly increase root mortality by depleting ST and SS reserves and indirectly inhibit the transport of photosynthetic products to roots (Hasibeder et al., 2014). Therefore, the results suggested that changes in NSCs caused by changes in environmental conditions were related to the variation in fine root morphological traits.

## Conclusion

This project was the first comprehensive investigation of the interactive response of root branch order and fine root NSC levels of *F. mandshurica* seedlings to drought intensity and soil substrate. The results confirmed that the variation in higher-order fine root biomass was higher than that in lower-order roots under different soil substrates and drought intensities. Second, the fine roots of the seedlings in the humus soil (first 5th root order) had a higher SS content and lower ST content. With increasing drought intensity, the SS and ST contents of the fine roots showed decreasing and increasing trends, respectively. The variation in fine root NSC content was related to the variation in root morphological traits (SRL, SRA, AD, and RTD) induced by drought and soil substrate rather than root chemical traits. This study reveals the adaptation strategies of *F. mandshurica* seedlings to drought under different soil substrate conditions, thereby enhancing the understanding of the construction and maintenance of the root system of *F. mandshurica*, and contributing to optimizing soil water management in *F. mandshurica* plantations. In further research, it is necessary to combine ^13^C-isotope-labeling technology to more deeply reveal the mechanism of carbohydrate distribution among different root branch orders under prolonged periods of drought.

## Data Availability Statement

The original contributions presented in the study are included in the article/Supplementary Material, further inquiries can be directed to the corresponding authors.

## Author Contributions

LJ and YY conceptualized the main question. LJ, JW, and ZL conducted the fieldwork. LJ and YL collected data and performed the data analyses. LJ wrote the manuscript. LJ, YY, and LZ revised the manuscript. All authors read and approved the manuscript.

## Conflict of Interest

The authors declare that the research was conducted in the absence of any commercial or financial relationships that could be construed as a potential conflict of interest.

## Publisher’s Note

All claims expressed in this article are solely those of the authors and do not necessarily represent those of their affiliated organizations, or those of the publisher, the editors and the reviewers. Any product that may be evaluated in this article, or claim that may be made by its manufacturer, is not guaranteed or endorsed by the publisher.
